# A fiery heart: case report of perimyocarditis in a patient with eosinophilic granulomatosis with polyangiitis

**DOI:** 10.1093/ehjcr/ytae414

**Published:** 2024-08-13

**Authors:** Dae Yong Park, Maria Isabel Planek, Abdul Khayyam Mohammed, Michael G Nanna, Tareq Alyousef

**Affiliations:** Section of Cardiovascular Medicine, Yale School of Medicine, 20 York Street, New Haven, CT 06510, USA; Division of Cardiology, Rush University Medical Center, 1620 W Harrison St, Chicago, IL 60612, USA; Department of Pathology, University of Illinois at Chicago, 840 S. Wood St, Chicago, IL 60612, USA; Section of Cardiovascular Medicine, Yale School of Medicine, 20 York Street, New Haven, CT 06510, USA; Division of Cardiology, Cook County Health, 1969 W. Ogden Ave, Chicago, IL 60612, USA

**Keywords:** Case report, EGPA, Vasculitis, Perimyocarditis, Eosinophilia, Non-dilated left ventricular cardiomyopathy

## Abstract

**Background:**

Eosinophilic granulomatosis with polyangiitis (EGPA) is a rare disease caused by small- to medium-sized vessel vasculitis which can also impact the heart. Because of its rarity and diverse clinical manifestations, diagnosis can be challenging. Here, we present a unique case of EGPA causing perimyocarditis in a young female patient.

**Case summary:**

A 37-year-old woman with hypertension, asthma, and sickle cell trait presented with palpitations, dyspnoea, and sharp chest pain. White blood cell was elevated to 16 300/μL with peripheral eosinophilia at 5216/μL. Electrocardiogram revealed sinus tachycardia with frequent non-sustained ventricular tachycardia. Echocardiogram showed an ejection fraction of 20–25% with severe diffuse hypokinesis and dilated cardiac chambers. Coronary angiogram was normal. Cardiac magnetic resonance imaging revealed focal subendocardial late gadolinium enhancement (LGE) of the septum and subepicardial LGE of the basal anterolateral wall of the left ventricle. Further work-up showed elevated Immunoglobulin E level, left antrochoanal polyp, and ground glass opacities in the left upper lobe. Endomyocardial biopsy showed interstitial infiltrates of eosinophils with sporadic necrosis, confirming the diagnosis of EGPA perimyocarditis. The patient was treated with prednisone, colchicine, and guideline-directed medical therapy.

**Discussion:**

This case report describes an unusual cause of perimyocarditis. Keeping a broad differential is important as diagnosis is challenging, and cardiac involvement in EGPA is associated with higher morbidity and mortality. Recognizing the typical manifestations of EGPA, implementing multidisciplinary approach, and promptly initiating appropriate treatment are crucial for the optimal management of EGPA perimyocarditis.

## Graphical Abstract

**Graphical Abstract:**
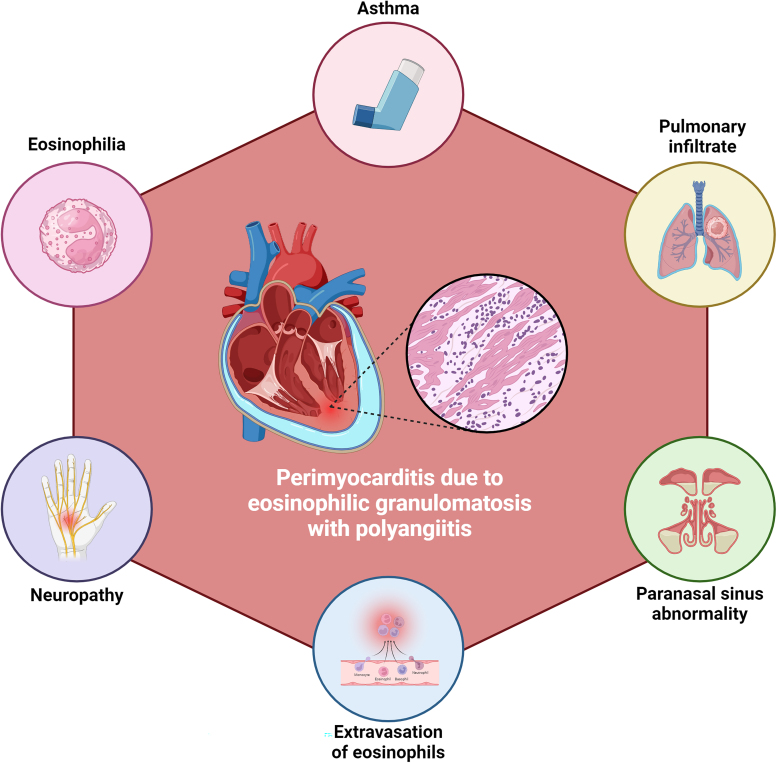
Characteristics of eosinophilic granulomatosis with polyangiitis that can hint towards its diagnosis in patients with perimyocarditis.

**Learning points**

- Make a differential of a rare aetiology of perimyocarditis.
- Understand the role of endomyocardial biopsy in the diagnosis.
- Promptly initiate a multidisciplinary approach to deliver the safest treatment with eosinophilic granulomatosis with polyangiitis impacting the heart.

## Introduction

Perimyocarditis secondary to eosinophilic granulomatosis with polyangiitis (EGPA) is a dangerous condition that can easily be missed because of its rarity.^1^ Early recognition and prompt introduction of multidisciplinary collaboration are required to better the prognosis of this multi-system vasculitis whose cardiac involvement is associated with a high mortality rate.^1,2^ Here, we report a rare case of perimyocarditis in a young female patient who initially presented with palpitations and dyspnoea and was subsequently diagnosed with EGPA.

## Case presentation

A 37-year-old African American woman, 5 months postpartum, with a medical history of hypertension, asthma, and sickle cell trait, arrived at the emergency department experiencing palpitations and dyspnoea since the day before. Normally, she could walk for 7–8 blocks, but upon arrival, she could only manage a few steps before feeling short of breath. The patient also mentioned experiencing orthopnoea, paroxysmal nocturnal dyspnoea, and malaise. At home, she took nifedipine 60 mg once daily, loratadine 10 mg as needed, and fluticasone inhaler twice daily, and there were no changes to her medications recently. Upon reaching the hospital, she complained of sharp, non-exertional chest pain behind her sternum, which intensified with inspiration and movement. On physical exam, the patient was normotensive but tachycardic and exhibited fine crackling sounds in both lower lung areas, but had no oedema in her extremities.

**Figure 1:**
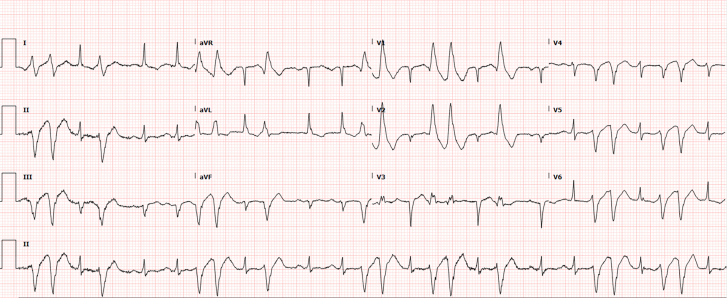
Electrocardiogram at admission. The electrocardiogram showed sinus tachycardia with frequent non-sustained ventricular tachycardia originating from the left ventricle. Q waves were also present in leads III and aVF. This is a right bundle branch superior axis ventricular tachycardia, consistent with origin of ventricular tachycardia from the anterior papillary muscle.

The white blood cell count was elevated to 16 300/μL with eosinophilic predominance at 32%. Troponin I was elevated to 5.183 ng/mL, and brain natriuretic peptide was 1099 pg/mL. Comprehensive metabolic panel, liver function tests, coagulation panel, and thyroid function tests were all within normal limits. Magnesium level was mildly decreased to 1.8 mg/dL. Urine toxicology was negative. Erythrocyte sedimentation rate and high-sensitivity C-reactive protein were elevated to 59 mm/h and 5.55 mg/dL, respectively. Electrocardiogram revealed sinus tachycardia with frequent runs of non-sustained ventricular tachycardia (NSVT) (*Figure 1*). Chest X-ray demonstrated moderate cardiomegaly with mild pulmonary vascular congestion.

**Figure 2:**
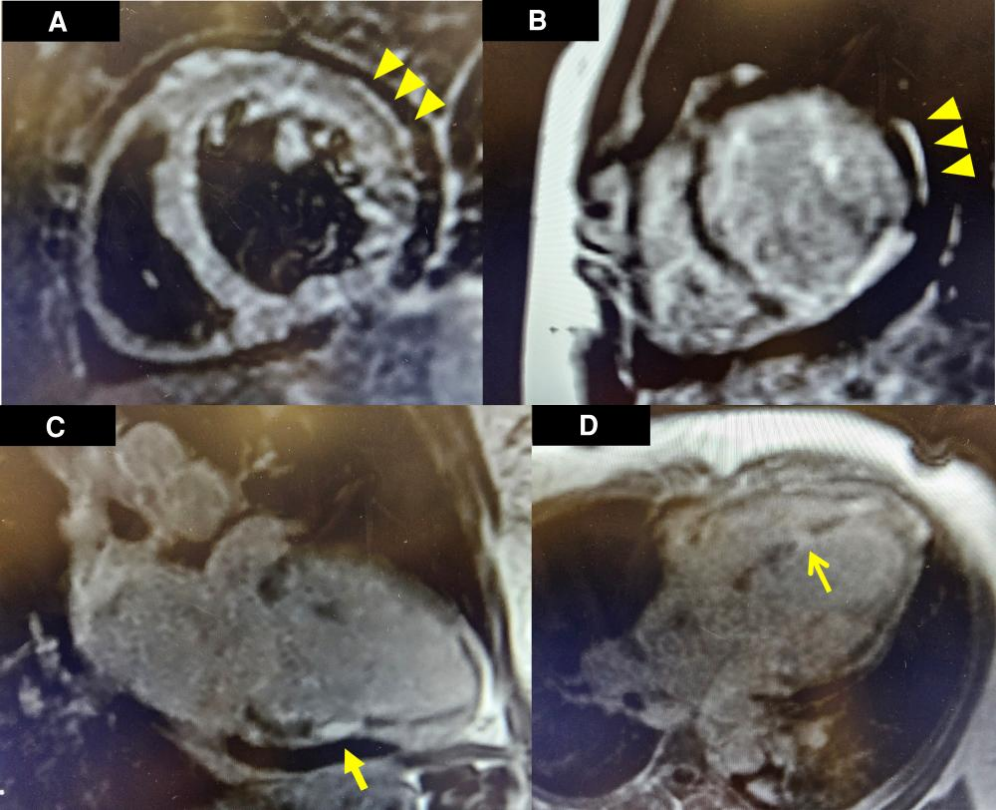
Cardiac magnetic resonance imaging findings. Subepicardial oedema can be visualized in the basal anterolateral segment of the left ventricle in T2 short-tau inversion recovery imaging (*A*). On post-contrast T1-prepared images, late gadolinium enhancement was observed in a corresponding and similar distribution of the basal anterolateral segment (*B*). Additional full thickness late gadolinium enhancement was noted in the midsegment of the inferior wall in a two-chamber view (*C*). Full thickness late gadolinium enhancement was noted in the mid inferoseptal segment of the left ventricle in a four-chamber view (*D*).

After receiving 4 g of intravenous (IV) magnesium sulfate, the patient’s NSVT resolved. Transthoracic echocardiogram revealed an ejection fraction of 20–25% with severe diffuse hypokinesis, dilatation of all cardiac chambers, and moderate free-flowing pericardial effusion, but no restrictive pattern (*Videos 1–3*; see Supplementary material online, *Video S1*). The coronary angiogram was normal (see Supplementary material online, *Figure S1*). She was given IV furosemide 40 mg twice daily and captopril 6.25 mg every 8 h. No inotrope was administered. High-dose aspirin and colchicine were started for presumed acute perimyocarditis. On the next day, cardiac magnetic resonance imaging revealed focal subendocardial late gadolinium enhancement (LGE) of the septum and subepicardial LGE of the basal anterolateral wall of the left ventricle (*Figure 2*). The presence of T2 hyperenhancement on the basal anterolateral wall indicated myocardial oedema, fulfilling the Lake Louise Criteria for acute myocarditis.^3^

**Figure 3:**
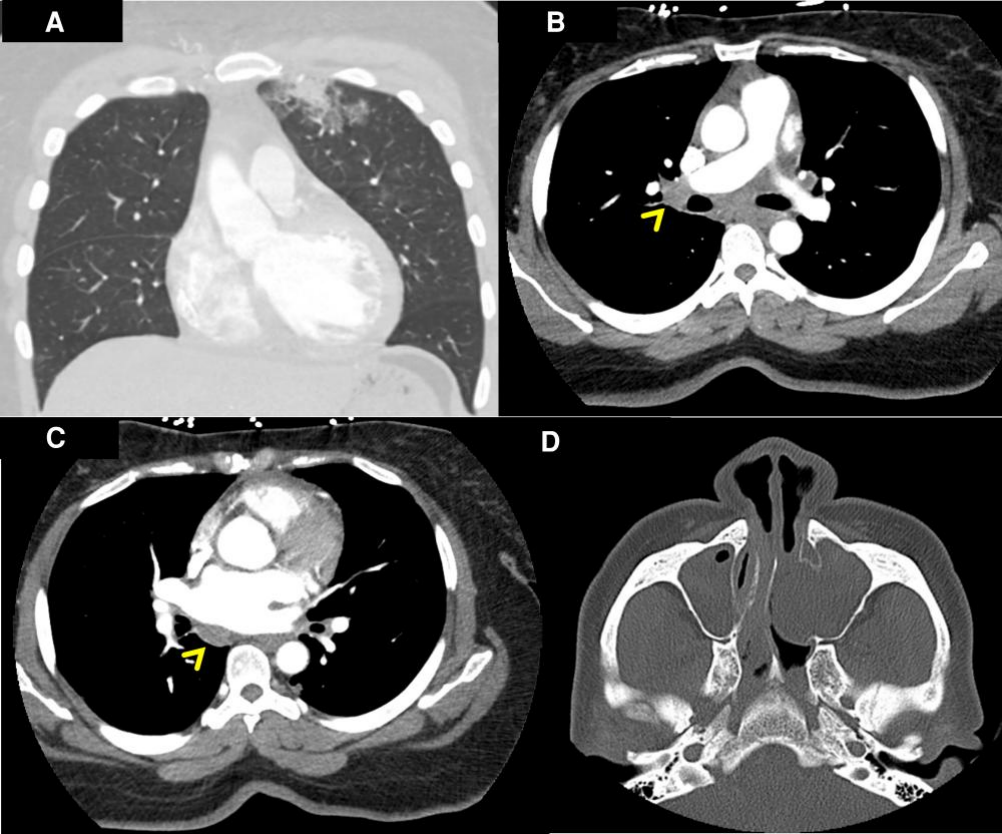
Computed tomography of the chest and sinuses. Patchy bilateral ground glass and airspace opacities were noted in the left upper lobe on computed tomography of the chest with intravenous contrast (*A*). Mediastinal lymphadenopathies were also visualized (linear arrowheads in *B* and *C*). Left antrochoanal polyp was found on computed tomography of the sinuses with intravenous contrast (*D*).

**Figure 4:**
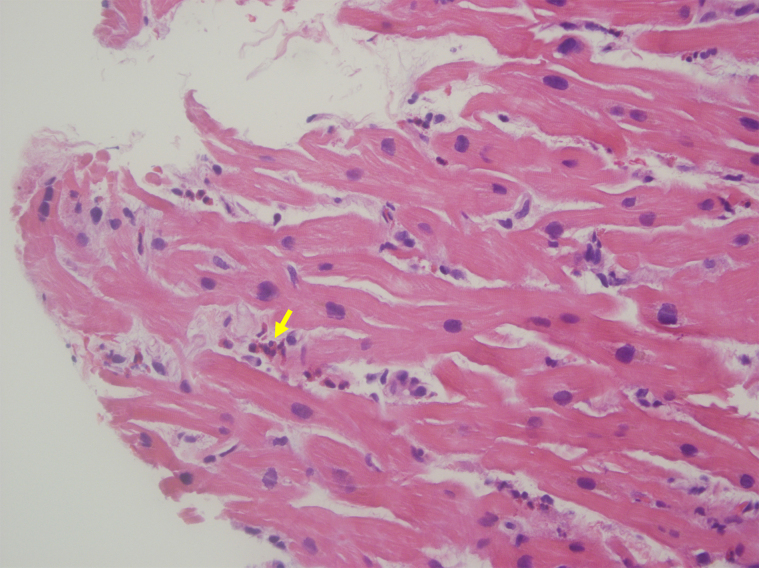
Endomyocardial biopsy. Haematoxylin and eosin stain of the endomyocardial biopsy shows infiltration of eosinophils (arrow) in the endomyocardium.

The patient’s dyspnoea improved, but she continued to experience intermittent chest pain. Upon further questioning, she revealed a history of recurrent sinusitis and nasopharyngitis. Patient tested negative for COVID-19, influenza, respiratory syncytial virus, and human immunodeficiency virus. Allergy and immunology and haematology specialists were consulted due to the presence of eosinophilia and concern for vasculitis. The patient’s Immunoglobulin G (IgE) level was found to be elevated to 758 kU/L, and a sinus computed tomography (CT) revealed a left antrochoanal polyp (*Figure 3*). Chest CT demonstrated mediastinal lymphadenopathy and patchy bilateral ground glass opacities in the left upper lobe. Antinuclear antibodies and antineutrophilic cytoplasmic antibodies were negative. Endomyocardial biopsy revealed interstitial infiltrates of eosinophils with sporadic myocyte necrosis (*Figure 4*). A nasal polyp biopsy showed reactive inflammation with prominent eosinophils, and a bone marrow biopsy revealed a blast count of 3% with marrow eosinophilia at 17%. Strongyloides IgE was negative, and bone marrow genetic tests on SCFD2, LNX, PDGFRA, PDGFRB, FGFR1, JAK2 V617F, CALR exon 9, JAK2 exon 12, MPL exon 10, and CSF3R exon 14/17 (genes associated with hypereosinophilic syndrome, eosinophilic leukaemia, and myeloproliferative neoplasms with eosinophilia) were all negative. The final diagnosis of EGPA was confirmed as the patient unequivocally met the diagnostic criteria outlined by the American College of Rheumatology (ACR).^4^

The patient received prednisone 80 mg daily, which led to resolution of the chest pain and eosinophilia. She was discharged home on prednisone, colchicine, and guideline-directed medical therapy with multidisciplinary follow-up. A follow-up echocardiogram performed 17 days after discharge revealed an improved ejection fraction of 40.

## Discussion

Eosinophilic granulomatosis with polyangiitis, formerly known as Churg–Strauss syndrome, is a rare autoimmune disease characterized by inflammation of small- and medium-sized blood vessels, leading to damage in various organs such as the lungs, skin, nerves, and gastrointestinal tract.^1^ Its prevalence ranges from 11 to 18 per million in the Western hemisphere, with about 16% afflicting the heart.^5,6^ While EGPA often presents with a triad of asthma, eosinophilia, and systemic vasculitis, its clinical spectrum can be diverse, making diagnosis challenging.^7^ The diagnosis was even more challenging in the presented case as initial differentials were broad, including postpartum cardiomyopathy, Loeffler syndrome, spontaneous coronary artery dissection, and genetic cardiomyopathies. Cardiac involvement in EGPA accounts for disproportionate morbidity and mortality, underscoring the importance of early recognition and prompt treatment.^7^ Cardiac manifestations can range from asymptomatic myocarditis to life-threatening conditions such as heart failure, myocardial infarction, or arrhythmias.^7^ In the presented case, the patient exhibited features of perimyocarditis and dilated cardiomyopathy. These findings highlight the importance of considering EGPA in the differential diagnosis of patients presenting with cardiac symptoms, particularly in those with a history of asthma, eosinophilia, and systemic vasculitis.

The diagnosis of EGPA relies on a combination of clinical, laboratory, and imaging findings. Peripheral eosinophilia is a hallmark feature, and elevated IgE levels can help differentiate EGPA from other forms of vasculitis.^8^ Chest X-rays often reveal pulmonary infiltrates, and CT scans can demonstrate sinusitis, lung nodules, or mediastinal lymphadenopathy.^8^ The ACR has established diagnostic criteria to aid in the identification of EGPA, requiring the presence of at least four of six criteria from asthma, eosinophilia, neuropathy, pulmonary infiltrates, paranasal sinus abnormality, and extravasation of eosinophils.^4^ In the presented case, the patient fulfilled all the ACR criteria except for neuropathy.^4^

Endomyocardial biopsy remains the gold standard for confirming the diagnosis of EGPA with cardiac involvement. Histopathological examination typically reveals interstitial infiltrates of eosinophils, myocyte injury, vasculitis, and granulomas.^9^ However, it is important to note that not all patients with EGPA exhibit these classic microscopic features, and the absence of granulomas or vasculitis does not exclude the diagnosis. Tissue involvement may be patchy or occur at a later stage of the disease.^1^

The management of EGPA involves a multidisciplinary approach, including cardiology, rheumatology, and haematology. Prompt initiation of treatment is essential to control disease activity, prevent organ damage, and improve outcomes. Glucocorticoids are the mainstay of therapy and have been shown to induce remission in most patients.^7^ However, achieving disease control and preventing relapse may require the use of additional immunosuppressive agents, such as methotrexate, azathioprine, mycophenolate mofetil, or rituximab.^7^

In conclusion, EGPA is a rare autoimmune disease with diverse clinical manifestations and multi-organ involvement. Early recognition of cardiac involvement in EGPA, which may require endomyocardial biopsy, is crucial for timely intervention and improved outcomes. Prompt initiation of glucocorticoid therapy is essential, and additional immunosuppressive agents may be necessary. Multidisciplinary efforts among healthcare professionals are vital for the optimal management of EGPA.

## Supplementary Material

ytae414_Supplementary_Data

## Data Availability

The data underlying this article will be shared on reasonable request to the corresponding author.

## Lead author biography

Dae Yong Park is currently a resident at the Department of Medicine at Cook County Health in Chicago, IL, USA. He is an aspiring academic cardiologist who is interested in diverse areas within cardiology. He is also a graduate student at the Master of Clinical Investigation at Northwestern University. His research interests pertain to clinical outcomes research, systematic reviews, and meta-analysis in various topics in cardiovascular medicine, including geriatric cardiology, coronary interventions, structural heart disease, and heart failure.
