# Supplementary material for: A fiery heart: case report of perimyocarditis in a patient with eosinophilic granulomatosis with polyangiitis
Source: Eur Heart J Case Rep. 2024 Aug 13;8(9):ytae414. doi: 10.1093/ehjcr/ytae414 (PMC11483628; doi:10.1093/ehjcr/ytae414)
Supplement: ytae414_Supplementary_Data [file ytae414_Supplementary_Data.zip › EGPA Perimyocarditis_Supplementary.pdf]

Figure S1. Coronary angiogram

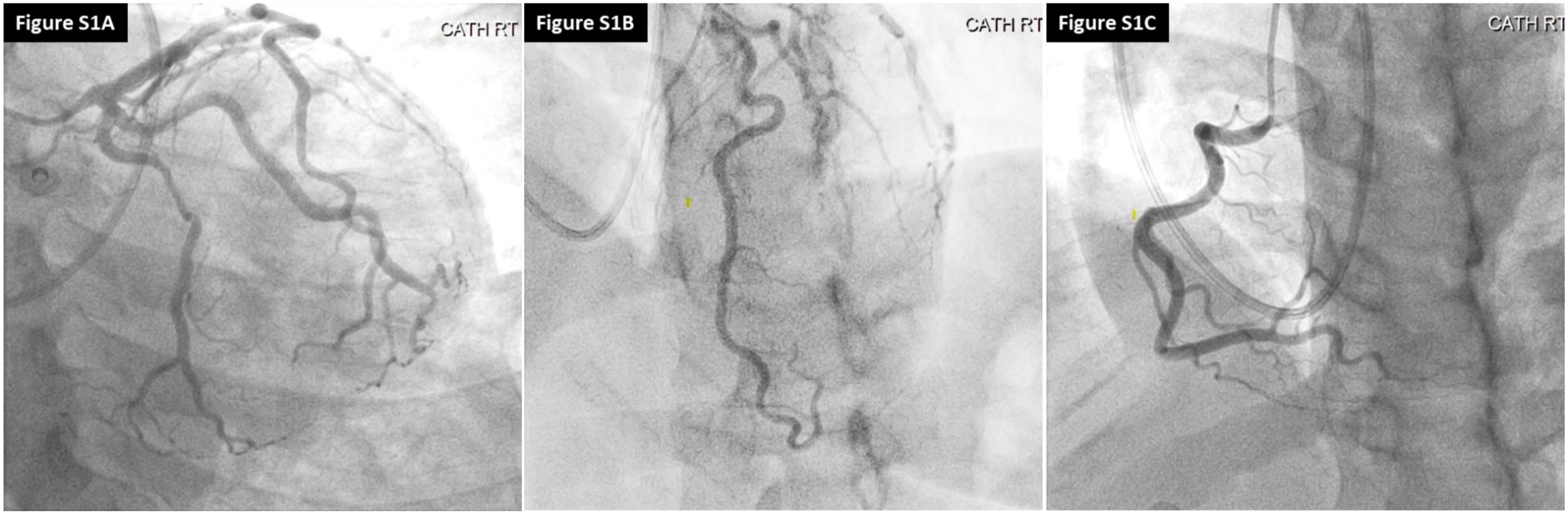

The left anterior oblique caudal (Figure S1A), left anterior oblique cranial (Figure S1B), and right coronary artery angiography (Figure S1C) show no obstruction in the patient’s coronary arteries.
